# Vagino-peritoneal fistula in pediatric patient with continuous ambulatory peritoneal dialysis and lupus nephropathy: An unusual case report

**DOI:** 10.1016/j.ijscr.2023.108167

**Published:** 2023-04-11

**Authors:** Gede Wirya Kusuma Duarsa, Made Dyah Vismita Indramila Duarsa, Pande Made Wisnu Tirtayasa, Bagus Ngurah Mahakrishna, Yenny Kandarini, Gusti Ayu Putu Nilawati

**Affiliations:** a Department of Urology, Sanglah General Hospital, Faculty of Medicine of Udayana University, Denpasar, Bali, Indonesia; b Faculty of Medicine, Udayana University, Bali, Indonesia; c Department of Child Health, Sanglah General Hospital, Denpasar, Bali, Indonesia; d Department of Internal Medicine, Sanglah General Hospital, Denpasar, Bali, Indonesia

**Keywords:** Continuous ambulatory peritoneal dialysis, Leakage peritoneal fluid, Lupus nephritis, Vagina peritoneal fistula

## Abstract

**Introduction and importance:**

Continuous Ambulatory Peritoneal Dialysis (CAPD) is one of the major renal replacement therapies that is widely used, especially in children. Although CAPD patients do not need to visit the hospital on a regular basis, it is frequently followed by complications such as peritonitis, hernia, pain, and, rarely, vagino-peritoneal fistula.

**Case presentation:**

A 13-year-old female was diagnosed with end-stage renal disease due to lupus nephritis and treated with Continuous Ambulatory Peritoneal Dialysis (CAPD) for three months. She underwent a laparoscopic diagnostic procedure and removal of CAPD after being diagnosed with vagino-peritoneal fistula with signs of recurrent peritonitis. During this time, patient has no symptoms regarding the SLE condition.

**Clinical discussion:**

Vagino-peritoneal fistula is one of the complications in CAPD patients that cause a decrease in patient's quality of life. The vaginal discharge might be an alarm sign for the possibility of a vagino-peritoneal fistula. The aggravation of inflammation due to the SLE condition may facilitate the perforation. Vagino-peritoneal fistula should be treated as soon as possible to prevent peritonitis.

**Conclusion:**

A rare finding of vagino-peritoneal fistula as a result of peritonitis due to CAPD could be aggravated by severe inflammation in SLE patients.

## Highlights

• A vagino-peritoneal fistula is indeed a rare case.
• Vaginal discharge in CAPD patients might be an alarm for vagino-peritoneal fistula.
• The perceived symptoms disturb the patient both psychologically and socially.
• Vaginal leakage after dialysis should be treated to prevent prolonged peritonitis.

## 1 Introduction

More than half of patients with Systemic Lupus Erythematous (SLE) have lupus nephritis, and up to 30 % of them may develop End-Stage Renal Disease (ESRD) [1]. Continuous Ambulatory Peritoneal Dialysis (CAPD) is one of the major renal replacement therapies that is widely used, especially in children. Although CAPD patients do not need to visit the hospital on a regular basis, it is frequently followed by complications such as peritonitis, hernia, pain, and, rarely, vagino-peritoneal fistula [2], [3]. Complications of vagino-peritoneal fistula do not directly cause mortality, but fistulas are one of the risk factors that cause a decrease in the patient's quality of life. A rare case of a 13-year-old female with ESRD due to lupus nephritis who had been previously treated with CAPD with multiple complications including recurrent peritonitis, CAPD malfunction, and vagino-peritoneal fistula was described in this report. To the best of our knowledge, this is the first study reporting vagino-peritoneal fistula in pediatric patient with CAPD who suffered from SLE. This case presentation followed SCARE Guideline Checklist 2020 [4].

## 2 Presentation of case

**Fig. 1:**
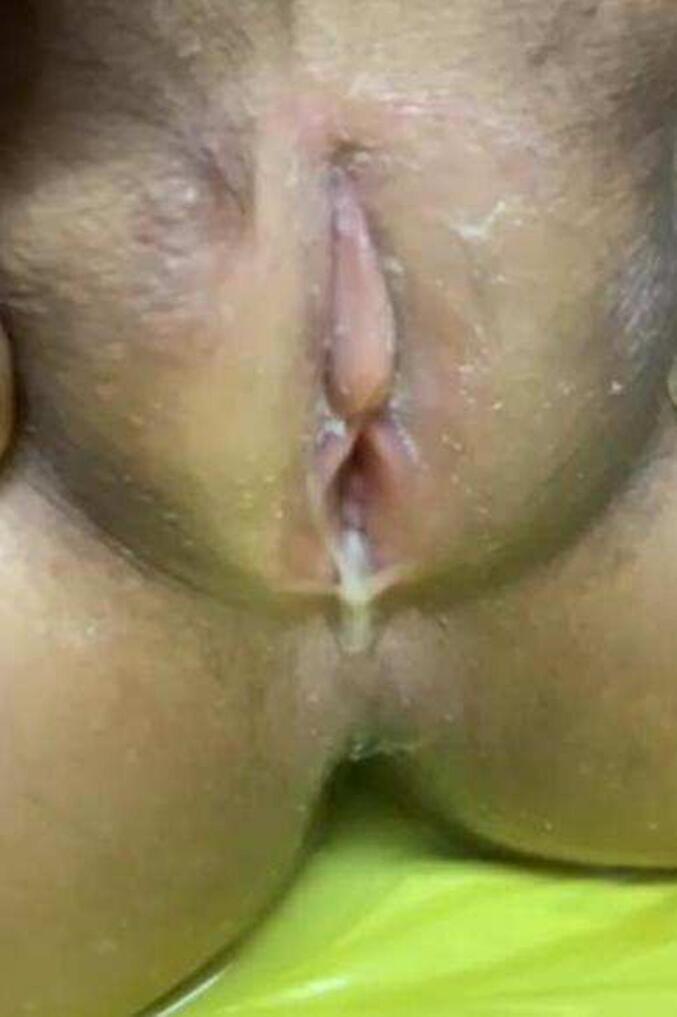
Purulent vaginal discharge one month after PD catheter repair.

**Fig. 2:**
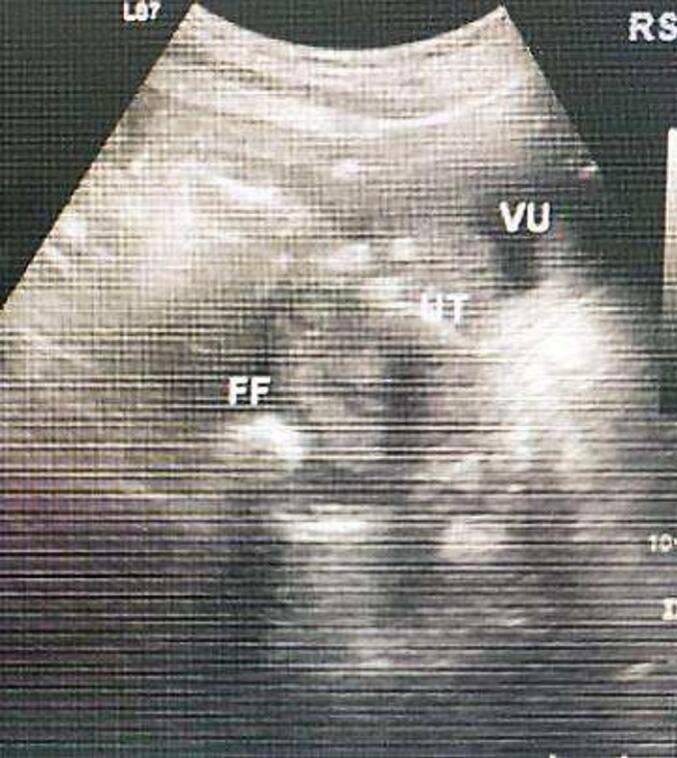
Free fluid in abdominal cavity found in abdominal ultrasound. FF: free fluid; UT: uterus; VU: vesical urinaria.

**Fig. 3:**
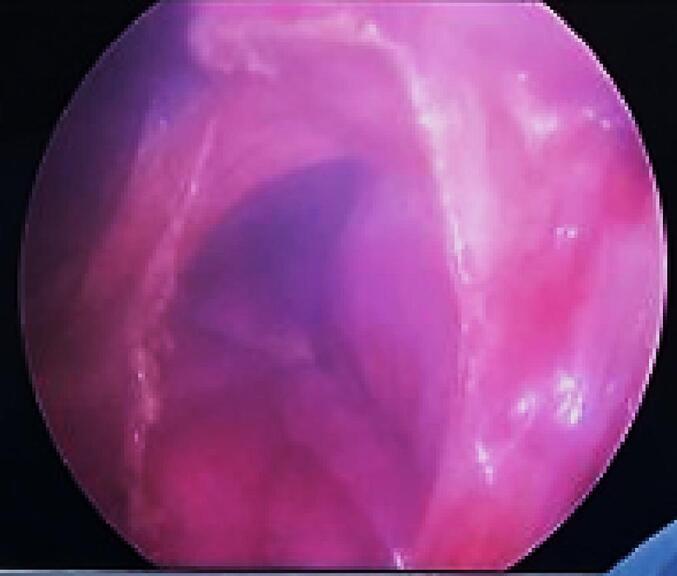
Severe adhesion during laparoscopic surgery.

A 13-year-old female with ESRD due to lupus nephritis. In regard to her age, the pediatrician suggested the use of CAPD. She underwent CAPD insertion and one-time repair afterward due to malfunction and peritonitis complications that occurred during treatment. After one month of the repair, she had a symptom of peritoneal dialysis (PD) fluid leak through the vagina when flushing the PD catheter. The discharge was collected with the result of a positive peritoneal cell (Fig. 1). The abdominal plain photo showed a normal PD catheter position. However, the abdominal ultrasound result showed free fluid in the abdominal cavity, suggesting a connection between the peritoneum and vagina (Fig. 2). During this time, patient has no symptoms regarding the SLE condition and the pediatrician did not give any medication at the mean time regarding Lupus condition. Furthermore, a laparoscopic diagnostic procedure was performed to assess the PD catheter and to detect the fistula. Pre-operative antibiotic was administered prior to surgery. During surgery, the fistula was difficult to find due to severe adhesion (Fig. 3). The team decided to flush normal saline into the peritoneal cavity and confirmed that there was fluid leakage coming out of the vagina since the urethra was attached to the urinary catheter. We decided to remove the PD catheter and plan for further evaluation of the fistula. After about fifty-three days of recuperation in the hospital, the patient returned home in stable condition without any complaints or any significant abnormal laboratory results. The patient's family refused to do further fistula examination. Two months after outpatient, the patient had no symptoms that suggested recurrence nor persistent vagino-peritoneal fistula. Patient is routinely controlled while undergoing hemodialysis.

## 3 Discussion

Internal leakage of PD fluid or fistula is a well-known complication of CAPD. The pathophysiology of fistulas in CAPD patients has not been described in detail, but in patients with a history of chronic systemic infection or prior episodes of peritonitis, fistulas may occur due to friable tissue. A vagino-peritoneal fistula is indeed a rare case, but based on the anatomical position that is sandwiched between the bladder and the rectum, a fistula through the vagina may form [2], [3], [5]. The case of vagino-peritoneal fistula in young female has been reported as early as 1983 as an unusual complication of peritoneal dialysis [6]. However, no previous study reported this case in pediatric patient with SLE.

The difficulty, in this case, is that because the patient is unmarried and still a virgin, no vaginal toucher or vaginal speculum examination was performed. Based on anamnesis, vaginal discharge increased, specifically when the PD catheter was flushed. The absence of true incontinence symptoms excludes the differential diagnosis of vesico-vaginal fistula in this patient. The patient had a history of chronic inflammatory conditions due to SLE for the last 3 years and prior episodes of peritonitis, so the patient was at risk for having friable tissue. However, patient did not show any flare regarding her SLE condition during examination. A history of surgery in the field of gynecology was denied. The bladder test did not reveal any fluid leakage that came out through the vagina when the bladder was passively filled through infusion. Biochemical confirmation showed positive peritoneal cell results. Abdominal ultrasound results also indicated the presence of free fluid in the abdominal cavity with a suspicious connection between the peritoneum and vagina. Eventually, this patient was diagnosed with a PD fluid leak through the vagina.

Diagnostic laparoscopic procedures were performed to confirm the location of the fistula and PD catheter. During the surgery, the fistula could not be found due to severe adhesion. Since the urethra was connected to a urinary catheter, it is possible to confirm the fistula that there was fluid leaking from the vagina by flushing normal saline into the peritoneal cavity. Exploration using vaginal speculum may necessary in this case. However, we did not perform vaginal exploration due to pediatric patient. The team decided to remove the PD catheter. Further examination is planned to evaluate the fistula while assessing the clinical condition of the patient after surgery. Currently, the patient's ESRD condition is managed using hemodialysis. Vagino-peritoneal fistula is an important diagnosis and should be recognized by the nephrologist whenever an unexplained vaginal discharge occurs in a female patient with CAPD. In particular present case, the aggravation of inflammation due to SLE condition may weaken the peritoneum wall and facilitated the perforation. Although the incidence is rare, the perceived symptoms disturb the patient both psychologically and socially. This case is unique in that vaginal leakage of dialysis fluid through a structurally normal genital tract is unknown. We suggest that patients with a vaginal leak from dialysis should be treated as soon as possible to prevent another prolonged peritonitis.

## 4 Conclusion

A rare finding of vagino-peritoneal fistula as a result of peritonitis due to CAPD could be aggravated by severe inflammation in SLE patients. There were no complaints or sequelae in patients with vagino-peritoneal fistula after laparoscopic diagnostics and removal of the PD catheter. This action provides good clinical outcomes for patients.

## Provenance and peer review

Not commissioned, externally peer-reviewed.

## Consent & ethics

The informed consent was written by the patient's parents in the Indonesian language for further publication of this case report and radiology images anonymously.

Written informed consent was obtained from the patient's parents/legal guardian for publication of this case report and accompanying images. A copy of the written consent is available for review by the Editor-in-Chief of this journal on request.

## Ethical approval

Patient's parents/legal guardian voluntarily consented to the study's publication, understanding that the patient's identity would remain private.

## Funding

None.

## Author contribution

Gede Wirya Kusuma Duarsa: Conceptualization. Made Dyah Vismita Indramila Duarsa, Pande Made Wisnu Tirtayasa: Writing Original draft. Bagus Ngurah Mahakrishna, and Gede Wirya Kusuma Duarsa: Reviewing and editing data curation. Yenny Kandarini, and Gusti Ayu Putu Nilawati: Supervision, Validation.

## Guarantor

Gede Wirya Kusuma Duarsa.

Made Dyah Vismita Indramila Duarsa.

## Research registration number

N/A.

## Declaration of competing interest

None.
